# Retinol-Binding Protein-4—A Predictor of Insulin Resistance and the Severity of Coronary Artery Disease in Type 2 Diabetes Patients with Coronary Artery Disease

**DOI:** 10.3390/biology10090858

**Published:** 2021-09-01

**Authors:** Sangeetha Perumalsamy, Wan Azman Wan Ahmad, Hasniza Zaman Huri

**Affiliations:** 1 Department of Clinical Pharmacy & Pharmacy Practice, Faculty of Pharmacy, Universiti Malaya, Kuala Lumpur 50603, Malaysia; geetha2_20@yahoo.com; 2 Cardiology Unit, Department of Medicine, Faculty of Medicine, Universiti Malaya, Kuala Lumpur 50603, Malaysia; wanazman@ummc.edu.my; 3 Clinical Investigation Centre (CIC), Universiti Malaya Medical Centre, Petaling Jaya 59100, Malaysia

**Keywords:** RBP-4, insulin resistance, severity of CAD, T2D, atherosclerotic CAD, endothelial dysfunction

## Abstract

**Simple Summary:**

Cytokines are cell-signaling molecules that cause cells to migrate to inflammation, infection, or trauma sites. An imbalance of cytokines in the body can result in severe illness. Increased cytokine retinol-binding protein-4 levels cause muscle, fat, and liver cells to become unresponsive to insulin and not absorb sugar from the blood. Type 2 diabetes, the most common type of diabetes, is caused by the unresponsiveness of insulin (insulin resistance). Moreover, elevated retinol-binding protein-4 causes fat and cholesterol buildup in the arteries of the heart. This results in coronary artery disease, a type of heart disease. These two diseases are hypothesized to share a common underlying cause, but the details have not been fully elucidated. Therefore, this study was conducted to find the association between retinol-binding protein-4 with insulin resistance and the severity of coronary artery disease. We postulated that retinol-binding protein-4 is linked to insulin resistance and the severity of coronary artery disease. This study proves a definitive relationship between retinol-binding protein-4 and insulin resistance and coronary artery disease severity. Hence, retinol-binding protein-4 may serve as a valuable biological indicator to depict insulin resistance and the severity of coronary artery disease.

**Abstract:**

(1) Background: Insulin resistance (IR) is the fundamental cause of type 2 diabetes (T2D), which leads to endothelial dysfunction and alters systemic lipid metabolism. The changes in the endothelium and lipid metabolism result in atherosclerotic coronary artery disease (CAD). In insulin-resistant and atherosclerotic CAD states, serum cytokine retinol-binding protein-4 (RBP-4) levels are elevated. The adipocyte-specific deletion of glucose transporter 4 (GLUT4) results in higher RBP-4 expression and IR and atherosclerotic CAD progression. (2) Aim: This study aimed to investigate the association of RBP-4 and clinical factors with IR and the severity of CAD. (3) Methods: Patients were recruited from diabetes and cardiology clinics and divided into three subgroups, namely (i) T2D patients with CAD, (ii) T2D-only patients, and (iii) CAD-only patients. The severity of CAD was classified as either single-vessel disease (SVD), double-vessel disease (DVD), or triple-vessel disease (TVD). An enzyme-linked immunosorbent assay was conducted to assess the concentration of serum RBP-4. Univariate (preliminary analysis) and multivariate (secondary analysis) logistic regressions were applied to assess the associations of RBP-4 and clinical factors with IR and the severity of CAD. (4) Results: Serum RBP-4 levels were associated with IR and the severity of CAD in all the three groups (all *p*-values are less than 0.05). Specifically, serum RBP-4 levels were associated with IR (*p* = 0.030) and the severity of CAD (SVD vs. DVD, *p* = 0.044; SVD vs. TVD, *p* = 0.036) in T2D patients with CAD. The clinical factors fasting plasma glucose (FPG) and angiotensin-converting-enzyme inhibitor (ACEI) were also associated with both IR and the severity of CAD in T2D patients with CAD. (5) Conclusion: RBP-4, FPG, and ACEI are predictors of IR and severity of CAD in T2D patients with CAD.

## 1. Introduction

Type 2 diabetes (T2D) has become a significant public health concern in developed and developing countries over the last few decades, making it a global health priority [1]. Insulin resistance (IR) is the fundamental key feature of T2D [2]. Although the mechanism of IR leading to atherosclerosis is not fully explicated, IR is said to be involved in endothelial dysfunction and alters systemic lipid metabolism, resulting in dyslipidemia and the well-known lipid triad of high levels of plasma triglycerides (TG), low levels of high-density lipoprotein (HDL), and high levels of low-density lipoproteins (LDL). This triad and endothelial dysfunction may lead to the formation of atherosclerotic plaques [3]. The formation of atherosclerotic plaques in the coronary arteries results in atherosclerotic coronary artery disease (CAD) [4]. The CAD is defined as severe when the atherosclerotic plaques narrow down the vessels with stenosis by more than 50% [5]. The severity is also classified on the basis of the number of vessels involved as a single-vessel disease (SVD), double-vessel disease (DVD), and triple-vessel disease (TVD). The most severe type of CAD is TVD with more than 50% stenosis, followed by DVD and SVD. DVD and TVD are also referred to as multivessel diseases [6].

In T2D patients, CAD is more complex, with small diffuse, calcified, multivessel involvement that frequently necessitates coronary revascularization besides optimal medical therapy to control angina [7]. Furthermore, CAD has been shown to increase mortality in T2D patients [8]. T2D and CAD are said to share the exact pathogenesis involving inflammation, endothelial dysfunction, and the release of proinflammatory cytokines [9]. Retinol-binding protein-4 (RBP-4) is a proinflammatory cytokine that may involve in the progression of IR, T2D, atherosclerosis and CAD. It is a 21 kDa cytokine produced predominantly in the liver and adipose tissue [10,11], which acts as an adipokine and fatty acid transporter that aid retinol (vitamin A) transport in the body [12]. It has recently been proposed that RBP-4-induced inflammation causes IR and CAD [13,14].

Although the role of RBP-4 in the pathogenesis of T2D and CAD is unknown, several studies have hypothesized that RBP-4 elevation causes IR and atherosclerotic CAD via the mitogen-activated protein kinase (MAPK) pathway [15,16,17]. Moreover, p44/42 MAPK, c-Jun N-terminal kinase, and p38 MAPK are all part of this pathway. When the p38 MAPK pathway is activated, glucose transporter 1 (GLUT1) expression increases, while glucose transporter 4 (GLUT4) expression decreases. The downregulation of GLUT4 expression leads to an increase in RBP-4 levels in the blood [18,19]. Chadt and Al-Hasani (2020) revealed that high RBP-4 secretion by adipocytes reduced GLUT4 expression in adipose tissue, as commonly reported in T2D patients [20]. In patients with CAD, there is an increase in epicardial RBP-4 and a decrease in GLUT4 levels [21].

Numerous studies have found elevated serum RBP-4 levels in T2D and CAD patients. RBP-4 levels of more than 55 g/mL were linked to an increased risk of T2D incidence in one study [22]. In another study, higher RBP-4 levels were linked to an increased risk of CAD, and RBP-4 levels increased as the number of stenosed vessels increased [23]. 

Because of both disorders sharing the same hypothesized mechanisms leading to endothelial dysfunction, and, thus, inflammation, RBP-4 can be the typical cytokine of IR and the severity of CAD in patients with T2D. In this study, we examined the relationship of RBP-4 and clinical factors with both IR and the severity of CAD in Malaysian T2D patients.

## 2. Materials and Methods

### 2.1. Participants

The selection of participants and inclusion and exclusion criteria were as earlier described (PMID: 34071097) [5]. This study used the same cohort as in the PMID: 34071097. Specifically, in this study, we investigate the cytokine (RBP-4) associations with IR and the severity of CAD, whereas PMID: 34071097 explored the genetic (rs17173608) associations with IR and the severity of CAD. The purpose of the study was explained to all participants, and they were asked to sign a written informed consent form. The patients were divided into three groups according to the presence of T2D with CAD, T2D without CAD, and CAD without T2D. 

### 2.2. Sample Size Calculation

The dichotomous test (PS software) was used to calculate the sample size under the assumptions of a level of significance, α of 0.05; the power of study at 0.80; the probability of exposure among controls, P_0_ at 0.65; and the probability of exposure among cases, P_1_ at 0.95 (the probability of exposure among cases was higher than of controls by 30%). The ratio of the control group to the case group, m was 1:1. As a result of the test, the minimum sample size required was 88 cases and 88 controls. There were 300 samples collected, namely 150 cases and 150 controls (150 T2D patients with CAD (cases); 90 T2D-only patients + 60 CAD-only patients (controls).

### 2.3. Aim and Hypotheses

The study aimed to investigate the association of RBP-4 and clinical factors with IR and the severity of CAD. Meanwhile, this study hypothesizes that RBP-4 is associated with IR and the severity of CAD in T2D patients with CAD and that clinical factors are associated with IR and the severity of CAD in T2D patients with CAD.

### 2.4. Demographic and Clinical Information, and Anthropometric Measurements

The assessments of demographic and clinical information and anthropometric measurements were as earlier described (PMID: 34071097) [5]. Laboratory investigation results, such as fasting plasma glucose (FPG), fasting plasma insulin (FPI), A1C, hs-CRP and lipid profile, the types and number of comorbidities, and details on the pharmacological treatments, were also obtained.

### 2.5. Biochemical Parameters

Biochemical parameters’ assessments were as earlier described (PMID: 34071097) [5]. Blood samples were taken after at least 8 h of fasting in the morning (7:00 a.m. to 10:00 a.m.). FPI and FPG were multiplied and divided by 22.5 to calculate the Homeostasis Model Assessment of Insulin Resistance (HOMA-IR) [23]. The concentrations of RBP-4 in the blood were determined by using an enzyme-linked immunosorbent assay (ELISA) kit and read at 450 nm with a microplate reader [24].

### 2.6. RBP-4 Assay Protocol

The samples were incubated for 30 min to an hour at room temperature. The blood samples were then centrifuged for 15 min at 1000× *g*. The serum was then drawn out and aliquoted. The serum was kept at −20 °C after the process. The serum samples were brought to room temperature before use. To avoid protein degradation and denaturalization, recurrent freeze–thaw cycles were avoided. For the RBP-4 ELISA assay, the quantitative sandwich enzyme immunoassay technique was used (Cusabio ELISA kit- Elabscience, Houston, TX, USA). The RBP-4 concentrations were measured in nanograms per milliliter.

### 2.7. Statistical Analysis

The preliminary and secondary analyses applied binary and multinomial logistic regression tests (univariate and multivariate), with age, race, BMI (body mass index), and gender adjusted as covariates. The association model of IR with RBP-4 levels and clinical factors was investigated by using binary logistic regression. Meanwhile, multinomial logistic regression analysis was used to evaluate the association model of the severity of CAD with RBP-4 levels and clinical factors. The secondary tests were based on the significant associations found in the preliminary analysis (*p* ≤ 0.05).

A plotted receiver-operating characteristic (ROC) curve was used to determine the cutoff point of IR and RBP-4 levels. To determine the optimal threshold of HOMA-IR and RBP-4 levels, the point on the ROC curve with the highest Youden index (sensitivity-(1-specificity)) and the point with the shortest distance from the point (0, 1) ((1-sensitivity)2 + (1-specificity)2) were calculated (cutoff point of HOMA-IR is 7.17 and RBP-4 levels are 1.6045 ng/mL).

### 2.8. Operational Definitions

Operational definitions were as earlier described (PMID: 34071097) [5].

## 3. Results

### 3.1. Demographic and Clinical Factors of Study Population

The results of demographic and clinical factors were as described earlier (PMID: 34071097) [5].

### 3.2. The Severity of CAD

**Figure 1:**
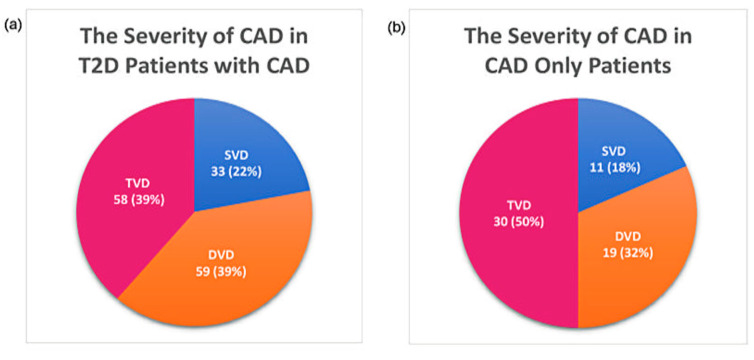
Distribution of patients according to the severity of CAD: (**a**) severity of CAD in T2D patients with CAD and (**b**) severity of CAD in CAD only patients.

Several T2D patients with CAD had multivessel disease (DVD, 39%, and TVD, 39%). Meanwhile, 50% of CAD patients had TVD. Figure 1 describes the distribution of patients according to the severity of CAD.

### 3.3. Association of Clinical Factors with RBP-4

Glycated hemoglobin (A1C) (*p* = 0.034), high-sensitive C-reactive protein (hs-CRP) (*p* < 0.001), low-density lipoprotein cholesterol (LDL-c) (*p* < 0.001), high-density lipoprotein cholesterol (HDL-c) (*p* = 0.001), triglycerides (TG) (*p* = 0.028), biguanides + insulin therapy (*p* < 0.001), biguanides + sodium–glucose co-transporter-2 (SGLT2) + insulin therapy (*p* = 0.001), biguanides + sulphonylureas (SU) combination therapy (*p* < 0.001), biguanides monotherapy (*p* < 0.001), SU monotherapy (*p* < 0.001), nitrates (*p* = 0.008), diuretics (*p* = 0.002), and cardiac glycosides (*p* < 0.001) were all significantly associated with RBP-4 levels in patients with T2D who presented with CAD. In T2D patients with CAD, cardiac glycoside was found to be 18.444 times most likely to be associated with RBP-4 levels. 

FPI and total cholesterol (TC) levels were significantly associated with RBP-4 levels in T2D-only patients. When comparing the two factors, the association of TC (OR = 1.345; *p* = 0.031) with RBP-4 levels in T2D-only patients was stronger than that of FPI (OR = 1.220; *p* = 0.032). In CAD-only patients, no significant associations were found between clinical factors and cytokines. Table 1 depicts the association of clinical factors of the study population with RBP-4 levels. Appendix A shows the significant results (*p*-values) of the preliminary associations.

### 3.4. Association of Clinical Factors with IR

In T2D patients with CAD, FPG (*p* = 0.011), FPI (*p* < 0.001), hs-CRP (*p* = 0.025), biguanides + dipeptidyl peptidase-4 inhibitor (DPP4i) + insulin treatment (*p* = 0.008), antiplatelet agents (*p* = 0.003), and angiotensin-converting-enzyme inhibitor (ACEI) (*p* = 0.026) were all linked to IR. The first-ranked factor was hs-CRP, associated with IR in T2D patients with CAD 2.378 times more likely. In the T2D-only group, FPI (*p* < 0.001) was significantly related to IR. FPG (*p* = 0.048) and FPI (*p* < 0.001) were linked to IR in CAD-only patients. FPG was the most powerful factor, being 2.570 times more likely to be associated with IR in CAD-only patients. Table 2 highlights the correlation of clinical factors to IR in this study population. Appendix A shows the significant results (*p*-values) of the preliminary associations.

### 3.5. Association of Clinical Factors with the Severity of CAD

In T2D patients with CAD, FPG (^a^ *p* = 0.007; ^b^ *p* = 0.012), FPI (^a^ *p* = 0.045), DPP4i (^a^ *p* < 0.001; ^b^ *p* < 0.001), SU + DPP4i (^a^ *p* = 0.011; ^b^ *p* = 0.016), biguanide + DPP4i + insulin (^a^ *p* < 0.001; ^b^ *p* = 0.003), fibrates (^b^ *p* < 0.001), statins (^b^ *p* = 0.018), ACEI (^a^ *p* = 0.032; ^b^ *p* = 0.029), alpha blockers (^a^ *p* = 0.008; ^b^ *p* = 0.020), and hematinic agents (^a^ *p* = 0.011; ^b^ *p* = 0.016) were significantly associated with the severity of CAD. In the CAD-only group, FPG (^a^ *p* < 0.001), FPI (^b^ *p* < 0.001), A1C (^b^ *p* < 0.001), LDL-c (^b^ *p* = 0.004, HDL-c (^a^ *p* < 0.001), and TG (^b^ *p* = 0.001) were associated with the severity of CAD. DPP4i was found to have the strongest association with the severity of CAD in T2D patients with CAD, with an OR value of 2.149. Table 3 demonstrates the association between clinical factors and the severity of CAD in the study population. Appendix A shows the significant results (*p*-values) of the preliminary associations

### 3.6. Association of RBP-4 Levels with IR and the Severity of CAD

**Figure 2:**
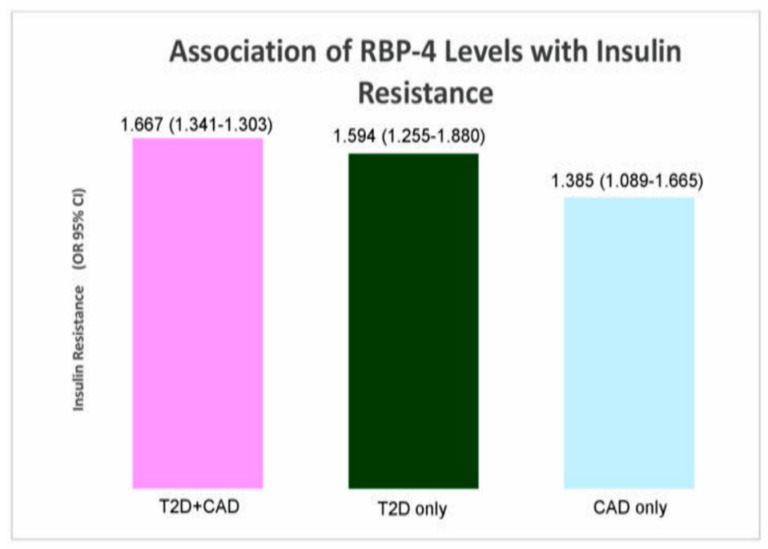
Association of RBP-4 levels with IR.

**Figure 3:**
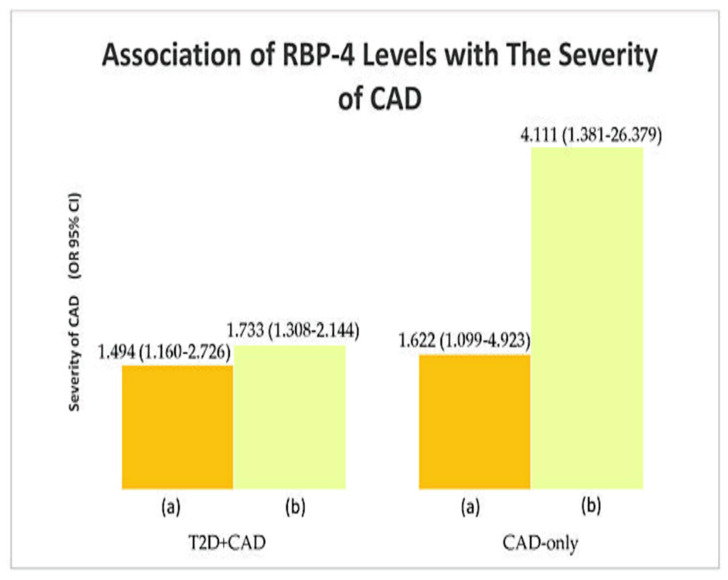
Association of RBP-4 levels with the severity of CAD: (**a**) DVD and (**b**) TVD; SVD is the reference group.

RBP-4 levels were associated with HOMA-IR levels (IR) in T2D patients with CAD (*p* = 0.002), T2D-only (*p* = 0.042), and CAD-only (*p* = 0.031) study population. RBP-4 levels were most associated with IR in T2D patients with CAD group (OR = 1.667). At the same time, significant associations were found between RBP-4 levels and the severity of CAD (T2D+CAD: ^a^ *p* = 0.017, ^b^ *p* = 0.022; CAD-only: ^a^ *p* = 0.002, ^b^ *p* = 0.001). RBP-4 levels formed the strongest association with severity of CAD in the CAD-only group (TVD vs. SVD); (OR = 4.111). Table 4, Figure 2,Figure 3 show the association of RBP-4 levels with IR and the severity of CAD. Appendix A shows the significant results (*p*-values) of the preliminary associations.

### 3.7. Association of IR and the Severity of CAD in Correlation with RBP-4 Levels and Clinical Factors (Secondary Analysis)

**Figure 4:**
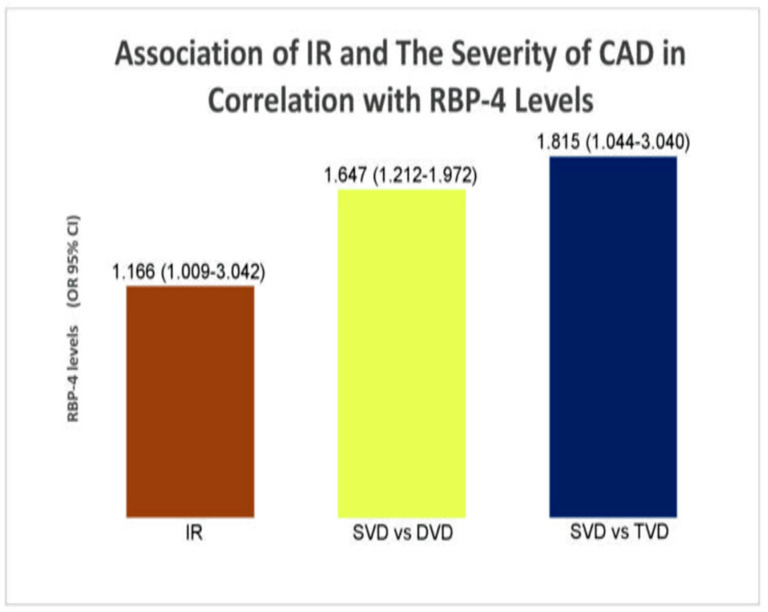
Association of IR and the severity of CAD in correlation with RBP-4 levels.

The significant variables from the preliminary analysis of the case group were used in the secondary analysis (T2D patients with CAD). FPG, ACEI, and RBP-4 were identified as predictors of IR in T2D patients with CAD, using a binary logistic regression model. In the multinomial analysis, the same factors were identified as predictors of the severity of CAD in T2D patients with CAD. Hence, FPG, ACEI, and RBP-4 were predictors of both IR and the severity of CAD in T2D patients with CAD. Table 5 and Figure 4 show the association of IR and the severity of CAD in correlation with RBP-4 levels. The secondary associations of IR and the severity of CAD in correlation with clinical factors were as earlier described (PMID: 34071097) [5].

## 4. Discussion

Elevated RBP-4 levels are known to be linked to IR, T2D, atherosclerosis, and CAD. Serum RBP-4 levels were significantly associated with A1C, hs-CRP, LDL-c, HDL-c, and TG in T2D patients with CAD in the preliminary associations. A previous study found that RBP-4 levels were significantly correlated with hs-CRP, LDL-c, and A1C with T2D and CAD, which is consistent with the current study’s findings [25]. At the same time, RBP-4 levels were also found to be associated with FPI and TC in T2D-only patients in this study. These findings are supported by studies conducted by Fan et al. (2019) and Wessel et al. (2019) [22,26]. 

Additionally, in their research, Ruijgrok et al. (2018) discovered a link between IR and FPG in their research [27]. Findings from the present study in the group of T2D patients with CAD concur with the findings of this study. This study found that FPI was associated with IR; however, no previous research on this association exists [28]. Furthermore, this study suggests FPI as a reliable and efficient test for detecting IR in people at risk of developing T2D and CAD. In a previous study, hs-CRP was correlated to IR [29]. In line with this, this study discovered an association between hs-CRP and IR. Moreover, hs-CRP is a marker that is used to determine the risk of heart disease. As inflammation appears to play a significant role in the pathogenesis of T2D and CAD, IR could have correlated with hs-CRP.

Moreover, biguanides + DPP4i + insulin treatment, antiplatelet agents, and ACEI were linked to IR in T2D patients with CAD. Thus far, no studies were conducted to investigate the relationship between IR and the mentioned OHA or pharmacological treatments in T2D patients with CAD.

In T2D-only patients, FPG was associated with IR. Khan et al. (2018) previously proposed a significant association between FPI and IR in T2D-only patients [30]. The results were comparable to the present study. Meanwhile, in the present study, FPI and FPG were significantly associated with IR in CAD-only patients. However, data are scarce on the correlation of FPI and FPG with IR in CAD-only patients. The present study’s findings revealed significant associations between FPG and FPI and the severity of CAD in T2D patients with CAD. Despite being on diabetic medications, patients with DVD and TVD had higher FPG and FPI levels. However, Srinivasan et al. (2017) discovered an association between hyperinsulinemia and adverse cardiac events in T2D patients [31]. 

A previous study found significant correlations between laboratory parameters, such as HDL-c, hs-CRP, and TG, and the severity of CAD [32,33]. This previous study’s findings differed from those of the present study. The hs-CRP levels in this study were within the normal range (< 1.0 mg/L). The successful reduction of hs-CRP levels in T2D patients with CAD was most likely due to the patients’ antihypertensive (ACEIs) and lipid-lowering (statins) pharmacological treatments. Previous studies had shown that antihypertensive and lipid-lowering medications lower hs-CRP levels [34]. Aside from hs-CRP, significant improvements in TC and LDL-c levels were observed. Despite improvements in hs-CRP and TG levels in the study group, no significant associations were found between the markers and the severity of CAD. These findings agree with the previous study by Razban et al. (2016) [34].

DPP4i was described as a second- or third-line add-on treatment that provided cardiovascular benefits without increasing the risk of heart failure, hypoglycemia, or death [35]. Besides DPP4i, statins are used to reduce the frequency of cardiovascular events in T2D patients with and without CAD [36]. Meanwhile, ACEIs are the first-line treatment for hypertension in T2D and CAD patients, and they have been shown to reduce the incidence and recurrence of atherosclerotic CAD [37]. According to previous research, fenofibrate may reduce CVD risk in T2D patients [38]. Although DPP4i and fibrates were significantly associated with DVD and TVD in T2D patients with CAD in this study, the associations were with patients who did not receive the medications. By contrast, 50.8% of patients with DVD and 56.9% of patients with TVD were taking ACEIs. Meanwhile, in the T2D patients with CAD group, statins were taken by 98.3% of DVD patients and 100% of TVD patients. A majority of T2D patients with CAD were not on DPP4i and fibrates, most likely because they were on first-line diabetic pharmacological treatments and insulin. Thereafter, a majority of the patients were taking ACEIs and statins because hypertension and dyslipidemia were the two most common comorbidities of T2D and CAD [5].

In the CAD-only group, FPG, FPI, HOMA-IR, A1C, hs-CRP, LDL-c, HDL-c, and TG were significantly associated with the severity of CAD. Hyperinsulinemia was identified as an independent risk factor for the severity of coronary artery stenosis in non-diabetic CAD patients by Srinivasan et al. (2017) [31]. Fasting serum insulin level was not associated with CAD stenosis in a study by Vafaeimanesh et al. (2018); however, an association was found between the two after the prescription of glucose [39]. According to the author’s knowledge, there have not been several studies on laboratory investigations associated with the severity of CAD in CAD-only patients. Hence, although the CAD-only patients are not diabetic, there are chances for them to develop T2D in the future as many of the participants of this group have higher readings of FPI, FPG, and A1C, and the CAD severity was associated with the mentioned clinical factors. 

In a previous study, serum RBP-4 levels were correlated to IR in T2D patients, as well as non-diabetic populations with a strong T2D family history [40]. At the same time, elevated serum RBP-4 was associated with metabolic syndrome components [41]. Previous research found a significant positive correlation between RBP-4 levels and CAD severity [23,42,43,44]. All of these studies support the present study’s outcomes. From the additional secondary analysis, we found that RBP-4 is associated with IR and the severity of CAD in T2D patients with CAD, together with the clinical factors FPG and ACEI [5]. Thus, increased RBP-4 levels in the patients may play an important role in the inflammatory progress and further development of IR and severe CAD.

This study shows that RBP-4 levels and clinical factors are related to IR and the severity of CAD in T2D patients with CAD in the Malaysian population. The elevated RBP-4 signifies the progression of IR and endothelial dysfunction. This could provide an initial clue to healthcare providers in optimizing the management or treatment of T2D and CAD patients. RBP-4 as an alternative to standard biomarkers, such as HOMA-IR and hs-CRP, could signal perhaps an early intervention to prevent the disease progression.

### Strengths and Limitations

One of the study’s major strengths is that the factors were examined by using a specific common cytokine, RBP-4, which was linked to both IR and the severity of CAD separately in previous studies. Furthermore, the study’s stringent inclusion and exclusion criteria provided the most effective means of reducing the effect of confounding variables. Another notable feature of this study is the use of sandwich ELISA (Cusabio Elabscience, Texas, USA). Compared with other ELISA methods, the sandwich ELISA method has the highest specificity because it involves two antibodies that detect different epitopes on the same antigen. It also has a high degree of flexibility and sensitivity. One inherent weakness of this study is that some variable data from the electronic medical records and the National Cardiovascular Disease Database were not available. Consequently, socioeconomic factors, lifestyle factors (such as diet logs), and medication adherence were not analyzed in this study. Furthermore, no questionnaires or interviews were used in this study to collect socioeconomic and lifestyle information from the participants.

## 5. Conclusions

RBP-4 was found to be significantly associated with IR and the severity of CAD in both preliminary and secondary analyses together with FPG and ACEI. Consequently, this study found that RBP-4 is a significant predictor of IR and the severity of CAD in T2D patients with CAD. This study suggests that identifying RBP-4 as a common predictor of IR and the severity of CAD in T2D patients with CAD may serve as a valuable clinical indicator to predict the progression of IR and the severity of CAD. This could prevent unnecessary clinical burden to the healthcare system.

## Figures and Tables

**Table 1:** Association of clinical factors of study population with RBP-4 levels.

Parameter	OR (95% CI)		
	T2D + CAD (*n* = 150)	T2D-Only (*n* = 90)	CAD-Only (*n* = 60)
FPG (mmol/L)	1.088 (0.983–1.204)	0.937 (0.825–1.065)	0.943 (0.328–2.707)
FPI (pmol/L)	0.995 (0.987–1.002)	**1.220 (1.041–1.430)**	0.936 (0.846–1.036)
A1C (%)	**0.797 (0.654–0.970)**	1.048 (0.856–1.283)	0.869 (0.268–2.820)
hs-CRP (mg/L)	**1.317 (1.052–1.632)**	1.728 (0.776–3.845)	1.214 (0.248–5.939)
TC (mmol/L)	1.017 (0.783–1.230)	**1.345 (1.033–1.589)**	1.476 (0.715–3.047)
LDL-c (mmol/L)	**2.918 (1.428–3.385)**	0.602 (0.339–1.069)	1.261 (0.533–2.986)
HDL-c (mmol/L)	**0.490 (0.300–0.800)**	1.181 (0.265–5.264)	0.965 (0.750–2.455)
TG (mmol/L)	**1.402 (1.201–1.702)**	0.740 (0.437–1.255)	0.983 (0.435–2.218)
Hypertension	0.244 (0.040–1.251)	1.102 (0.175–6.940)	3.062 (0.248–7.879)
Dyslipidemia	2.336 (0.708–7.708)	2.800 (0.548–14.311)	0.557 (0.096–3.244)
Peripheral neuropathy	0.635 (0.325–1.240)	1.021 (0.440–2.368)	5.667 (0.239–19.655)
Chronic kidney disease (CKD)	1.615 (0.691–3.778)	1.483 (0.526–4.183)	0.160 (0.009–2.823)
Retinopathy	0.547 (0.255–1.173)	0.839 (0.338–2.083)	5.667 (0.778–10.661)
Anemia	0.783 (0.048–12.760)	0.731 (0.050–2.065)	4.076 (0.421–40.755)
Gastritis	0.786 (0.333–1.546)	4.371 (0.437–43.763)	0.327 (0.026–4.034)
Biguanides	**6.400 (2.024–20.237)**	0.613 (0.220–1.705)	–
Sulphonylureas	**17.714 (5.812–53.993)**	1.482 (0.590–3.722)	–
DPP4i	8.400 (0.056–34.855)	1.005 (0.399–2.529)	–
AGI	0.786 (0.045–1.433)	0.731 (0.088–4.033)	–
Meglitinides	0.786 (0.088–2.113)	1.031 (0.326–3.264)	–
Biguanide + SU	**10.000 (3.240–30.866)**	1.360 (0.539–3.432)	–
SU + DPP4i	9.056 (0.076–65.877)	0.907 (0.144–5.715)	–
Biguanide + insulin	**1.206 (1.093–1.458)**	–	–
Biguanide + SU + insulin	1.290 (0.310–5.365)	–	–
Biguanide + DPP4i + insulin	0.021 (0.006–0.043)	–	–
Biguanide + SGLT2 + insulin	**1.161 (1.053–1.493)**	–	–
SGLT2 + insulin	0.308 (0.034–2.821)	–	–
Antiplatelet agents	4.114 (0.469–36.102)	–	0.008 (0.002–0.015)
ACEI	0.847 (0.443–1.619)	0.724 (0.268–1.951)	0.429 (0.090–2.043)
ARB II	0.530 (0.237–1.187)	1.052 (0.429–2.579)	2.909 (0.666–12.708)
Calcium channel blockers	1.041 (0.496–2.182)	1.235 (0.534–2.859)	1.818 (0.390–8.466)
Beta blockers	1.106 (0.578–2.114)	1.029 (0.445–2.377)	0.318 (0.064–1.574)
Alpha blockers	9.664 (0.099–45.123)	0.731 0.045–3.912)	–
Nitrates	**2.657 (1.331–5.303)**	–	2.629 (0.425–16.263)
Fibrates	7.690 (0.065–33.878)	–	–
Statins	0.040 (0.012–0.077)	7.760 (0.013–27.112)	–
Diuretics	**1.297 (1.141–1.624)**	1.283 (0.522–3.153)	2.629 (0.425–16.263)
Antianginal drugs	1.983 (0.935–4.205)	–	0.500 (0.046–5.423)
Hematinic agents	3.925 (0.402–38.903)	0.676 (0.059–7.735)	0.380 (0.061–2.354)
Cardiac glycosides	18.444 (2.331–145.961)	–	–

Computed using binary logistic regression analysis. Bold font indicates significance at *p* < 0.05. OR, odds ratio; CI, confidence interval. The cutoff point of RBP-4 was used in this analysis (<1.604 ng/mL>); ‘–’ indicates not relevant. FPG, fasting plasma glucose; FPI, fasting plasma insulin; A1C, glycated hemoglobin; hs-CRP, high-sensitive C-reactive protein; LDL-c, low-density lipoprotein cholesterol; HDL-c, high-density lipoprotein cholesterol; TG, triglycerides; TC, total cholesterol; ACEI, angiotensin-converting-enzyme inhibitor; AGI, alpha-glucosidase inhibitors; ARB II, angiotensin II receptor blockers; DPP4i, dipeptidyl peptidase-4 inhibitor; SGLT2, sodium–glucose co-transporter-2; SU, sulphonylureas.

**Table 2:** Association of clinical factors with IR in study population.

Parameter	HOMA-IR, OR (95% CI)		
	T2D + CAD (*n* = 150)	T2D-Only (*n* = 90)	CAD-Only (*n* = 60)
FPG (mmol/L)	**1.160 (1.031–1.306)**	1.010 (0.894–1.142)	**2.570 (1.097–5.773)**
FPI (pmol/L)	**1.233 (1.146–1.327)**	**1.376 (1.197–1.581)**	**1.368 (1.167–1.603)**
A1C (%)	1.102 (0.913–1.331)	1.098 (0.896–1.344)	2.122 (0.759–5.932)
hs-CRP (mg/L)	**2.378 (1.155–4.899)**	1.394 (0.636–3.057)	3.502 (0.909–13.493)
TC (mmol/L)	0.813 (0.622–1.063)	1.159 (0.809–1.660)	0.775 (0.443–1.356)
LDL-c (mmol/L)	0.942 (0.650–1.366)	1.119 (0.675–1.855)	0.806 (0.405–1.606)
HDL-c (mmol/L)	0.738 (0.500–1.089)	0.702 (0.159–3.100)	0.524 (0.053–5.140)
TG (mmol/L)	0.973 (0.673–1.407)	0.799 (0.482–1.324)	0.627 (0.292–1.342)
Hypertension	0.614 (0.147–2.556)	1.687 (0.268–10.617)	6.304 (0.044–19.030)
Dyslipidemia	0.515 (0.176–1.505)	1.951 (0.456–8.341)	0.684 (0.149–3.134)
Peripheral neuropathy	0.633 (0.319–1.258)	0.980 (0.426–2.253)	–
Chronic kidney disease (CKD)	0.990 (0.415–2.359)	2.645 (0.893–7.831)	2.867 (0.169–48.744)
Retinopathy	1.319 (0.629–2.768)	0.879 (0.360–2.147)	–
Anemia	1.596 (0.098–26.032)	–	–
Gastritis	–	1.098 (0.148–8.152)	–
Biguanides	0.750 (0.365–1.540)	0.689 (0.254–1.870)	–
Sulphonylureas	1.076 (0.496–2.332)	0.714 (0.292–1.747)	–
DPP4i	0.006 (0.002–0.010)	1.133 (0.455–2.822)	–
AGI	1.586 (0.066–2.887)	0.915 (0.052–4.835)	–
Biguanide + SU	1.201 (0.509–2.835)	0.413 (0.163–1.046)	–
SU + DPP4i	0.012 (0.006–0.030)	4.718 (0.506–43.984)	–
Biguanide + insulin	0.681 (0.333–1.394)	–	–
Biguanide + SU + insulin	1.630 (0.391–6.787)	–	–
Biguanide + DPP4i + insulin	**1.860 (1.043–2.027)**	–	–
Biguanide + SGLT2 + insulin	1.033 (0.445–2.395)	–	–
SGLT2 + insulin	2.455 (0.398–15.155)	–	–
Antiplatelet agents	**1.454 (1.032–1.895)**	–	6.011 (0.042–36.772)
ACEI	**1.444 (1.227–1.868)**	2.353 (0.872–6.351)	3.800 (1.006–14.351)
ARB II	1.829 (0.845–3.961)	0.524 (0.214–1.285)	0.429 (0.123–1.495)
Calcium channel blockers	0.662 (0.303–1.446)	0.644 (0.280–1.481)	1.768 (0.488–6.397)
Beta blockers	0.824 (0.426–1.594)	1.318 (0.575–3.024)	2.111 (0.509–8.751)
Alpha blockers	2.702 (0.038–16.806)	–	0.364 (0.033–1.452)
Nitrates	0.768 (0.382–1.544)	–	0.433 (0.086–2.196)
Fibrates	2.607 (0.028–6.442)	–	–
Statins	0.009 (0.004–0.014)	15.436 (0.078–66.022)	–
Diuretics	1.699 (0.853–3.384)	0.750 (0.305–1.843)	0.433 (0.086–2.195)
Antianginal drugs	0.594 (0.267–1.320)	–	9.923 (0.950–33.701)
Hematinic agents	1.607 (0.220–11.735)	0.536 (0.047–6.128)	2.308 (0.456–11.690)
Cardiac glycosides	0.263 (0.058–1.232)	–	–

Computed using binary logistic regression analysis. Bold font indicates significance at *p* < 0.05. OR, odds ratio; CI, confidence interval. HOMA-IR cutoff point: 7.17; ‘–’ indicates not relevant. FPG, fasting plasma glucose; FPI, fasting plasma insulin; A1C, glycated hemoglobin; hs-CRP, high-sensitive C-reactive protein; LDL-c, low-density lipoprotein cholesterol; HDL-c, high-density lipoprotein cholesterol; TG, triglycerides; TC, total cholesterol; ACEI, angiotensin-converting-enzyme inhibitor; AGI, alpha-glucosidase inhibitors; ARB II, angiotensin II receptor blockers; DPP4i, dipeptidyl peptidase-4 inhibitor; SGLT2, sodium–glucose co-transporter-2; SU, sulphonylureas.

**Table 3:** Association between clinical factors and the severity of CAD in study population.

Parameter	Severity of CAD, OR (95% CI)	
	T2D + CAD (*n* = 150)	CAD-Only (*n* = 60)
FPG (mmol/L)	**^a^** ** 1.815 (1.710** **–** **1.935)** **^b^ 1.875 (1.771** **–** **1.992)**	**^a^1.651 (1.201–2.110)** ^b^ 0.458 (0.151–1.388)
FPI (pmol/L)	^a^ 1.011 (0.997–1.026) **^b^ 1.015 (1.001–1.030)**	^a^ 0.984 (0.885–1.094) **^b^ 1.553 (1.054–2.105)**
A1C (%)	^a^ 0.898 (0.712–1.133) ^b^ 0.939 (0.747–1.179)	^a^ 0.626 (0.161–2.444) **^b^ 1.318 (1.087–1.858)**
hs-CRP (mg/L)	^a^ 0.652 (0.369–1.154) ^b^ 0.801 (0.518–1.237)	^a^ 3.229 (0.548–19.036) ^b^ 2.726 (0.526–14.473)
TC (mmol/L)	^a^ 0.880 (0.632–1.227) ^b^ 0.772 (0.546–1.092)	^a^ 0.685 (0.314–1.492) ^b^ 1.424 (0.712–2.848)
LDL-c (mmol/L)	^a^ 1.169 (0.722–1.895) ^b^ 0.901 (0.543–1.494)	^a^ 0.510 (0.197–1.321) **^b^ 1.722 (1.296–2.538)**
HDL-c (mmol/L)	^a^ 1.007 (0.602–1.686) ^b^ 1.059 (0.635–1.764)	**^a^3.754 (1.185–76.172)** ^b^ 2.218 (0.130–3.789)
TG (mmol/L)	^a^ 1.090 (0.689–1.724) ^b^ 0.726 (0.437–1.207)	^a^ 0.736 (0.285–1.899) **^b^ 1.299 (1.007–1.523)**
Hypertension	^a^ 2.963 (0.331–26.504) ^b^ 1.143 (0.100–13.105)	^a^ 4.144 (0.349–20.714) ^b^ 5.329 (0.957–29.532)
Dyslipidemia	^a^ 0.926 (0.207–4.147) ^b^ 1.373 (0.330–5.711)	^a^ 1.875 (0.171–20.609) ^b^ 2.000 (0.207–19.336)
Peripheral neuropathy	^a^ 0.783 (0.320–1.912) ^b^ 0.660 (0.271–1.609)	– –
Chronic kidney disease (CKD)	^a^ 0.875 (0.272–2.818) ^b^ 0.763 (0.240–2.424	^a^ 0.055 (0.015–0.368) ^b^ 0.976 (0.843–1.280)
Retinopathy	^a^ 0.791 (0.298–2.097) ^b^ 1.006 (0.371–2.728)	– –
Anemia	^a^ 1.006 (0.954–1.087) ^b^ 1.014 (0.076–1.632)	^a^ 1.065 (0.045–2.060) ^b^ 0.063 (0.036–0.123)
Gastritis	– –	^a^ 1.800 (0.101–31.988) ^b^ 2.900 (0.166–50.815)
Biguanides	^a^ 1.203 (0.491–2.945) ^b^ 1.500 (0.602–3.740)	– –
Sulphonylureas	^a^ 1.008 (0.387–2.625) ^b^ 1.800 (0.646–5.018)	– –
DPP4i	**^a^** ** 1.269 (1.008** **–** **1.865)** **^b^ 2.149 (1.320** **–** **3.326)**	– –
Biguanide + SU	^a^ 1.319 (0.450–3.872) ^b^ 1.466 (0.490–4.387)	– –
SU + DPP4i	**^a^** ** 1.654 (1.054** **–** **2.022)** **^b^ 1.754 (1.132** **–** **2.503)**	– –
Biguanide + insulin	^a^ 0.525 (0.194–1.418) ^b^ 0.441 (0.164–1.184)	– –
Biguanide + SU + insulin	^a^ 2.850 (0.451–17.999) ^b^ 1.833 (0.348–9.652)	– –
Biguanide + DPP4i + insulin	**^a^** ** 1.545 (1.008** **–** **1.967)** **^b^ 1.877 (1.210** **–** **3.116)**	– –
Biguanide + SGLT2 + insulin	^a^ 1.778 (0.612–5.165) ^b^ 1.367 (0.487–3.837)	– –
SGLT2 + insulin	^a^ 0.891 (0.078–10.210) ^b^ 0.875 (0.076–10.033)	– –
Antiplatelet agents	^a^ 1.123 (0.098–12.872) ^b^ 1.745 (0.174–17.492)	^a^ 4.647 (0.077–8.945) ^b^ 0.945 (0.768–1.490)
ACEI	**^a^** ** 1.487 (1.085** **–** **3.532)** **^b^ 1.166 (1.032** **–** **2.890)**	^a^ 0.833 (0.126–5.504) ^b^ 0.889 (0.151–5.241)
ARB II	^a^ 1.253 (0.453–3.468) ^b^ 1.006 (0.371–2.728)	^a^ 1.607 (0.255–10.132) ^b^ 1.636 (0.289–9.255)
Calcium channel blockers	^a^ 0.567 (0.209–1.537) ^b^ 1.032 (0.362–2.946)	^a^ 1.200 (0.182–7.926) ^b^ 1.636 (0.289–9.255)
Beta blockers	^a^ 0.703 (0.299–1.654) ^b^ 0.548 (0.231–1.299)	^a^ 0.375 (0.036–3.865) ^b^ 0.500 (0.052–4.834)
Alpha blockers	**^a^** ** 1.795 (1.076** **–** **4.644)** **^b^ 1.900 (1.056** **–** **3.012)**	– –
Nitrates	^a^ 1.153 (0.485–2.742) ^b^ 2.112 (0.853–5.230)	^a^ 1.875 (0.171–20.609) ^b^ 1.111 (0.103–11.965)
Fibrates	^a^ 0.029 (0.006–0.144) **^b^ 1.056 (1.008–1.768)**	– –
Statins	^a^ 25.265 (0.122–46.004) **^b^ 1.087 (1.004–1.255)**	– –
Diuretics	^a^ 0.975 (0.395–2.404) ^b^ 0.950 (0.385–2.346)	^a^ 16.240 (3.209–57.778) ^b^ 13.325 (1.620–25.921)
Antianginal drugs	^a^ 0.729 (0.276–1.923) ^b^ 1.367 (0.487–3.837)	– –
Hematinic agents	**^a^** ** 1.540 (1.021** **–** **2.006)** **^b^ 1.444 (1.058** **–** **2.244)**	^a^ 0.533 (0.049–5.862) ^b^ 0.900 (0.084–9.692)
Cardiac glycosides	^a^ 1.025 (0.277–3.797) ^b^ 3.862 (0.667–22.350)	– –

Computed using multinomial logistic regression analysis. Bold font indicates significance at *p* < 0.05. OR: odds ratio; CI: confidence interval. Severity of CAD: ^a^ DVD, ^b^ TVD, and SVD is the reference group; ‘–’ indicates not relevant. FPG, fasting plasma glucose; FPI, fasting plasma insulin; A1C, glycated hemoglobin; hs-CRP, high-sensitive C-reactive protein; LDL-c, low-density lipoprotein cholesterol; HDL-c, high-density lipoprotein cholesterol; TG, triglycerides; TC, total cholesterol; ACEI, angiotensin-converting-enzyme inhibitor; AGI, alpha-glucosidase inhibitors; ARB II, angiotensin II receptor blockers; DPP4i, dipeptidyl peptidase-4 inhibitor; SGLT2, sodium–glucose co-transporter-2; SU, sulphonylureas.

**Table 4:** Association of RBP-4 levels with IR and the severity of CAD.

Parameter	OR (95% CI)		
	T2D + CAD (*n* = 150)	T2D-Only (*n* = 90)	CAD-Only (*n* = 60)
Insulin resistance	**1.667 (1.341** **–** 1.303) *	**1.594 (1.255** **–** 1.880) *	**1.385 (1.089** **–** 1.665) *
Severity of CAD	**^a^** ** 1.494 (1.160** **–** **2.726) ^¥^** **^b^ 1.733 (1.308** **–** **2.144) ^¥^**	–	**^a^** ** 1.622 (1.099** **–** **4.923) ^¥^** **^b^ 4.111 (1.381** **–** **26.379) ^¥^**

* Computed using binary logistic regression analysis. ^¥^ Computed using multinomial logistic regression analysis. HOMA-IR cutoff point: 7.17. Bold font indicates significance at *p* < 0.05. Insulin-sensitive (IS) is used as the reference group (IS vs. IR) for insulin resistance. SVD was used as the reference group (^a^ SVD vs. DVD; ^b^ SVD vs. TVD) for the severity of CAD. Adjusted for the covariates age, race, gender, and BMI. OR, odds ratio; CI, confidence interval; ‘–’ indicates not relevant.

**Table 5:** Association of IR and the severity of CAD in correlation with RBP-4 levels.

Parameter	OR (95% CI)	*p*-Value
Insulin resistance	1.166 (1.009–3.042) *	**0.030**
Severity of CAD	^a^ 1.647 (1.212–1.972) ^¥^ ^b^ 1.815 (1.044–3.040) ^¥^	**0.044** **0.036**

* Computed using binary logistic regression analysis. ^¥^ Computed using multinomial logistic regression analysis. HOMA-IR cutoff point: 7.17. Bold font indicates significance at *p* < 0.05. Insulin-sensitive (IS) is used as the reference group (IS vs. IR) for insulin resistance. SVD was used as the reference group (^a^ SVD vs. DVD; ^b^ SVD vs. TVD) for the severity of CAD, adjusted for the covariates age, race, gender, and BMI. OR, odds ratio; CI, confidence interval.

## Data Availability

The data presented in this study are available on request from the corresponding author. The data are not publicly available as they contain information that could compromise the privacy of research participants.

## Appendix A

**Table A1:** Summarization of significant outcomes from preliminary analysis of study (*p* < 0.05).

Association of RBP-4 with Clinical Factors		
Group	Parameter	*p*-Value
T2D + CAD	A1C	0.034
	hs-CRP	<0.001
	LDL-c	<0.001
	HDL-c	0.001
	TG	0.028
	Bi + I	<0.001
	Bi + SGLT2 + I	0.001
	Bi + SU	<0.001
	Bi	<0.001
	SU	<0.001
	Nitrates	0.008
	Diuretics	0.002
	Cardiac glycosides	<0.001
T2D-only	FPI	0.032
	TC	0.031
CAD-only	–	–
Association of clinical factors with IR and the severity of CAD		
Group	Factor	*p*-value
T2D + CAD		
IR	FPG	0.011
	FPI	<0.001
	hs-CRP	0.025
	Bi + DPP4i + I	0.008
	Antiplatelet agents	0.003
	ACEI	0.026
Severity of CAD	FPG	^a^ 0.007 ^b^ 0.012
	FPI	^a^ 0.045
	DPP4i	^a^ <0.001 ^b^ <0.001
	SU + DPP4i	^a^ 0.011 ^b^ 0.016
	Bi + DPP4i + I	^a^ <0.001 ^b^ 0.003
	ACEI	^a^ 0.032 ^b^ 0.029
	AB	^a^ 0.008 ^b^ 0.020
	Fibrates	^b^ <0.001
	Statins	^b^ 0.018
	Hematinic agents	^a^ 0.011 ^b^ 0.016
T2D-only		
IR	FPI	<0.001
Severity of CAD	–	–
CAD-only		
IR	FPG	0.048
	FPI	<0.001
Severity of CAD	FPG	^a^ <0.001
	FPI	^b^ <0.001
	A1C	^b^ <0.001
	LDL-c	^b^ 0.004
	HDL-c	^a^ <0.001
	TG	^b^ 0.001
Association of RBP-4 with IR and the severity of CAD		
Group	Factor	*p*-value
T2D + CAD		
IR	RBP-4	0.002
Severity of CAD		^a^ 0.017 ^b^ 0.022
T2D-only		
IR	RBP-4	0.042
Severity of CAD		–
CAD-only		
IR	RBP-4	0.031
Severity of CAD		^a^ 0.002 ^b^ 0.001

Association of clinical factors and RBP-4 with IR, association of clinical factors with RBP-4 computed by using binary logistic regression analysis. Association of clinical factors and RBP-4 with the severity of CAD computed using multinomial logistic regression analysis. SVD was used as the reference group (^a^ SVD vs. DVD; ^b^ SVD vs. TVD); ‘–’ indicates not relevant.
